# Comparison between Roux-en-Y gastrojejunostomy and Billroth-II with Braun anastomosis following partial gastrectomy: A randomized controlled trial

**DOI:** 10.1016/j.amsu.2022.103544

**Published:** 2022-03-28

**Authors:** Azita Shishegar, Matin Vahedi, Fereshteh Kamani, Mehrdad Fathi Kazerouni, Morteza Aghajanpour Pasha, Farhad Fathi

**Affiliations:** a Department of Surgery, Faculty of Medicine, AJA University of Medical Sciences, Tehran, Iran; b Department of Surgery, Taleghani Hospital, Shahid Beheshti University of Medical Sciences, Tehran, Iran; c Department of Thoracic Surgery, Imam Khomeini Hospital Complex, Tehran University of Medical Sciences, Iran; d Department of Internal Medicine, AJA University of Medical Sciences, Tehran, Iran

**Keywords:** Gastrectomy, Reconstruction, Billroth, Braun, Roux en Y anastomosis

## Abstract

Partial gastrectomy surgeries are conducted frequently due to various reasons but there is no consensus regarding the method of choice for gastrointestinal reconstruction. In this study we aimed to compare Billroth II with Braun anastomosis and Roux-en-Y gastrojejunostomy. This randomized, Single-blind clinical trial was conducted on patients presented to surgery clinic at Besat and Taleghani hospital between 2018 and 2020 who were gastrectomy candidates. Patients undergoing Billroth II reconstruction with Braun anastomosis or Roux-en-Y gastrojejunostomy were enrolled in the study. Demographics, operation durative and intraoperative blood loss, early postoperative complications, re-admission within 30 days post operation, 30-day and 90-day mortality were recorded. at an at least 3-month post-surgery period, patients were followed for late postoperative complications. If necessary, endoscopy was conducted and biopsy was taken. Analysis was performed with SPSS version 22. A total of 84 patients in two 42-patient groups were evaluated. All parameters were the same in two groups except operation duration and intraoperative bleeding (significantly higher in RY group), food residue (significantly higher in RY group) and bile in remnant stomach (significantly higher in B2B group). These two methods of reconstruction are comparable in terms of postoperative complications and mortality rates. Food residue and bile reflux are two determinants which should be kept in mind when choosing the surgery plan by surgeons.

This trial was prospectively registered in the Iranian Registry of Clinical Trials with a registration ID of IRCT20130706013875N3.

## Highlights

• Roux-en-Y gastrojejunostomy and Billroth-II with Braun anastomosis are comparable in terms of postoperative complications.
• Roux-en-Y gastrojejunostomy and Billroth-II with Braun anastomosis are comparable in terms of mortality rates.
• Food residue and bile reflux are two determinants which should be kept in mind when choosing the surgery plan by surgeons.

## 1 Introduction

Each year, around one million cases of gastric cancer are diagnosed worldwide. Gastric cancer is the 5th most commonly diagnosed cancer in the world [1]. Globally, gastric cancer ranks third among causes of cancer-related deaths [2]. With advances in earlier diagnosis of gastric cancer, rates of partial or total gastrectomy as the curative treatment have increased. The important issue after partial gastrectomy is reconstruction of gastrointestinal tract to preserve continuity of GI system [3]. Three procedures of Billroth-I (BI), Billroth-II (BII) and Roux-en-Y (RY) have been considered as the main reconstruction surgeries [3]. Bile reflux and esophagitis, dumping syndrome and delayed gastric emptying have been reported as the most common post-gastrectomy complications [[4], [5], [6], [7]]. Reflux gastritis after BII is a serious complication which impacts patients’ quality of life. Reflux gastritis itself is a potential risk factor for gastric cancer [[8], [9], [10]]. With introduction of Roux-en-Y gastrojejunostomy, risk of reflux gastritis declined but an increase in incidence of Roux syndrome due to stasis in Roux arm was reported. Technically, Roux-en-Y is more complicated and time-consuming compared to BII. Internal hernia is also reported to be higher after Roux-en-Y reconstruction [[11], [12], [13]]. To reduce the risk of bile gastritis in BII method, Braun enteroenterostomy has been introduced which decreases the flow of bile into the stomach [14]. Thus, BII combined with Braun anastomosis has been regarded as an alternative for RY. In this study, we aimed to compare the outcomes and complications of Roux-en-Y gastrojejunostomy and Billroth II with Braun anastomosis for reconstruction following partial gastrectomy surgeries.

## 2 Material and methods

### 2.1 Study design and patients

This randomized, Single-blind clinical trial was conducted between January 2018 to January 2020 in Besat and Taleghani hospitals of Tehran. Study sample included all the patients who needed gastrectomy referred to the surgery clinic. All patients were taken informed consent before enrollment in the study. Patients undergoing Billroth II and Braun anastomosis (B2B) or Roux-en-Y gastrojejunostomy (RY) after gastrectomy were included in our study. Patients with ages of <18 or >75 years, simultaneous malignancies, end-stage renal disease, severe or fulminant hepatic failure, severe psychiatric diseases, reconstruction procedures other than Billroth II and Braun anastomosis/Roux-en-Y gastrojejunostomy or unwilling to continue with study steps were excluded. Operations were performed by two expert surgeons who had performed at least 250 reconstruction surgeries.

This trial is conducted and reported in line with the CONSORT criteria, and the entire checklist is submitted as an attachment (http://www.consort-statement.org/). This trial was prospectively registered in the Iranian Registry of Clinical Trials with a registration ID of IRCT20130706013875N3 (https://www.irct.ir/trial/34389).

Demographics, comorbidities, pre-operative serum albumin, surgery duration, type of surgery, the reason for surgery, blood transfusion requirement during surgery, postoperative infection, intraoperative abscess, anastomotic leak, postoperative hospital stay, postoperative pneumonia, deep venous thrombosis, re-admission within 30 days' post-surgery, 30-day mortality and 90-day mortality were documented. Patients’ follow-up was performed at least 3 months after surgery via telephone or visits. GerdQ questionnaire was also used for evaluation of reflux symptoms in patients.

Patients were also assessed for dumping syndrome, diarrhea, delayed emptying, obstruction symptoms or re-admission. In symptomatic patients, endoscopic evaluation was suggested. Patients who accepted to undergo endoscopy, were fasting for 12 h. Endoscopic assessment was done for evidences of esophagitis, gastritis, mass in remnant stomach, anastomotic stricture, food residue in stomach and bile in stomach. In cases of any pathology in endoscopy, biopsies were taken for histopathologic examination.

### 2.2 Interventions: surgical techniques

To perform Billroth II and Braun anastomosis, a antecolic loop from jejunum and a 40-cm afferent loop for gastrojejunostomy were used. Anastomosis was done in a two-layer manner with absorbable continuous sutures in inner layer and nonabsorbable suture was used for outer layer. Afterwards, side-to-side anastomosis was performed at 25 cm distal to gastrojejunostomy between efferent and afferent loops in two layers. For Roux-en-Y reconstruction, Roux limb divided 20 cm distal to the ligament of Treitz and gastrojejunostomy was performed between the jejunum and remnant stomach. Then, an end-to-side jejunojejunostomy was done between the limbs approximately 40 cm distal to the gastrojejunostomy.

### 2.3 Outcomes

The outcome measure of the study included:

1) Gastroesophageal Reflux 3 months after surgery, which was measured by GerdQ questionnaire
2) Dumping syndrome 3 months after surgery, which was measured by medical history taking and examination
3) obstruction and delayed emptying 3 months after surgery, which was measured by medical history taking and examination
4) Gastritis 3 months after surgery, which was measured by endoscopy
5) Esophagitis 3 months after surgery, which was measured by endoscopy
6) Anastomosis site stenosis 3 months after surgery, which was measured by endoscopy

### 2.4 Sample size

*GerdQ questionnaire was used to calculate sample size based on previous studies.* Total sample size of the trial was 80 patients *(40 in each arm), considering a power of 80%,* a 2-sided significance level of 5%, and an attrition rate of 20%.

### 2.5 Randomization and blinding

Individuals were randomly divided into two groups based on randomized block randomization method. Randomized blocks were used for this purpose. A random sample based on this method from Sealedenvelope.com was extracted. None of the patients were aware of the technique of their surgery, but the surgeon and the researcher were aware of the type of technique for each patient.

### 2.6 Statistical analysis

Data analysis was performed with SPSS version 22. Mean and standard deviation, frequency and percentage were provided for descriptive analysis. To compare groups, student t-test was used for quantitative parameters. For qualitative parameters, chi-square test or Fischer's exact test were used. Statistical significance level was considered as p-value <0.05.

## 3 Results

A total of 84 patients in two 42-patient groups were analyzed. Demographic and baseline characteristics show no significant differences between groups (p-value> 0.05) but intraoperative parameters of operation duration and intraoperative blood loss reveal significantly higher surgery duration and blood loss in RY group compared to B2B patients. The details of baseline characteristics and intraoperative parameters are listed in Table 1, Table 2.

**Table 1:** Demographics and baseline characteristics in patients of B2B and RY groups.

Parameter	B2B	RY	p-value
Age (years)	59.8 ± 12.7	57.3 ± 11.8	0.299
Gender			
- Male	26 (61.9%)	31 (73.80%)	0.721
- Female	16 (38.09%)	11 (26.19%)	
Preoperative weight (kg)	62.3 ± 9.9	66.4 ± 12.3	0.251
Preoperative BMI (kg/m^2^)	22.7 ± 2.1	23.9 ± 3.3	0.339
ASA class			
- I	24 (57.14%)	27 (64.28%)	0.209
- II	18 (42.85%)	15 (35.71%)	
Preoperative albumin (mg)	3.5 ± 0.4	3.6 ± 0.2	0.711
Comorbidities			
- Renal failure	1 (2.38%)	1 (2.38%)	0.961
- Liver failure	3 (7.14%)	1 (2.38%)	0.127
- Diabetes mellitus	8 (19.04%)	10 (23.80%)	0.341
Surgery indication			
- Peptic ulcer	5 (11.90%)	6 (14.28%)	0.347
- GI bleeding	8 (19.04%)	5 (11.90%)	
- Cancer	29 (69.04%)	31 (73.80%)

**Table 2:** Intraoperative parameters in B2B and RY groups.

Parameter	B2B	RY	p-value
Surgery duration (min)	141.7 ± 32.9	166.3 ± 27.9	0.007
Blood loss (ml)	160.3 ± 52.3	252.7 ± 62.9	0.021

Patients’ weight 3 months after surgery was 57.1 ± 9.2 kg in B2B group and 59.6 ± 11.1 kg in RY group (p-value = 0.871). GerdQ score was 6.90 ± 1.2 and 5.40 ± 1.37 in B2B and RY groups, respectively (p = 0.722). Thus, both groups show no remarkable difference in terms of weight and quality of life three months after operation.

Early postoperative complications did not show significant difference between groups. The details are summarized in Table 3. Re-operation was performed in 3 patients (7.14%) and 2 patients (4.76%) of B2B and RY groups, respectively (p = 0.411). Re-admission within 30 days after operation occurred in 3 patients (7.14%) of B2b group and 3 patients (7.14%) of RY group (p = 0.882). 30-day mortality was observed in 1 patient (2.3%) and 1 patient (2.3%) of B2B and RY groups, respectively (p = 0.597).

**Table 3:** Early postoperative complications in B2B and RY groups.

Complication	B2B	RY	p-value
Surgical site infection	2 (4.7%)	3 (7.14%)	0.312
Anastomotic leak	1 (2.3%)	2 (4.76%)	0.844
GI bleeding	2 (4.7%)	1 (2.3%)	0.571
Pneumonia	2 (4.7%)	3 (7.14%)	0.568
Deep venous thrombosis	1 (2.3%)	1 (2.3%)	0.471
Renal failure requiring dialysis	1 (2.3%)	1 (2.3%)	0.398
Myocardial infarction	1 (2.3%)	1 (2.3%)	0.254
Adjuant therapy requirement	22 (52.3%)	19 (45.2%)	0.891
Postoperative bleeding	1 (2.3%)	1 (2.3%)	0.254

Late complications at 3 months after operation also showed no significant difference between two surgical procedures. The details of these complications are presented in Table 4. 3-month re-admission rate was seen in 5 patients (11.9%) of B2B group and 4 patients (9.52%) of RY group (p = 0.221). 3-month mortality occurred in 2 patients (4.7%) in each of B2B and RY groups (p = 0.613).

**Table 4:** Late postoperative complications in B2B and RY groups.

Complication	B2B	RY	p-value
Diarrhea	3 (7.14%)	4 (9.52%)	0.209
Dumping syndrome	2 (4.7%)	1 (2.3%)	0.811
Dysphagia	4 (9.52%)	3 (7.14%)	0.742
Odynophagia	2 (4.7%)	2 (4.7%)	0.671
Delayed gastric emptying	5 (11.9%)	4 (9.52%)	0.339
Obstruction symptoms	2 (4.7%)	1 (2.3%)	0.592

Fourteen patients (33.33%) in B2B group and 11 patients (26.19%) in RY group underwent endoscopic evaluation within 6 months after operation. The details of endoscopic findings are demonstrated in Table 5.

**Table 5:** Endoscopic findings in B2B and RY groups.

Finding	B2B	RY	p-value
Esophagitis			
- A	7 (16.6%)	6 (14.28%)	0.254
- B	1 (2.3%)	1 (2.3%)	
- C	1 (2.3%)	1 (2.3%)	
- D	0 (0%)	0 (0%)	
Gastritis			
- Mild	8 (19.04%)	5 (11.90%)	0.511
- Moderate	2 (4.76%)	2 (4.76%)	
- Severe	2 (4.76%)	0 (0%)	
Erosion in stomach	1 (2.3%)	0 (0%)	0.594
Mass in stomach	0 (0%)	0 (0%)	1.00
Anastomotic stricture	1 (2.3%)	1 (2.3%)	0.679
Food residue			
- 1	0 (0%)	2 (4.7%)	0.034
- 2	0 (0%)	2 (4.7%)	
- 3	0 (0%)	1 (2.3%)	
- 4	0 (0%)	0 (0%)	
Bile in stomach	8 (19.04%)	1 (2.3%)	0.012

Histopathologic evaluation of biopsies taken in endoscopy revealed no statistically significant difference between groups. Metaplasia showed relatively higher rates in RY group compared to B2B group but still it was not significant (p = 0.081). The details of histopathologic findings are presented in Table 6.

**Table 6:** Histopathologic findings in biopsies of patients.

Finding	B2B	RY	p-value
Chronic inflammation	9 (21.42%)	7 (16.66%)	0.581
Neutrophilic infiltration	7 (16.66%)	7 (16.66%)	0.239
Metaplasia	1 (2.37%)	4 (9.52%)	0.081
Glandular atrophy	0 (0%)	0 (0%)	1.00
*Helicobacter pylori*	2 (4.7%)	5 (11.90%)	0.821
Dysplasia	0 (0%)	0 (0%)	1.00

## 4 Discussion

This study was focused on comparison of Billroth II and Braun anastomosis with Roux-en-Y gastrojejunostomy as reconstruction procedures after partial gastrectomy. We found out that there was no difference between these procedures in terms of patients' characteristics, outcomes and complications except for operation duration and blood loss which were significantly higher in Roux-en-Y gastrojejunostomy. Despite the increase in number of partial gastrectomies performed around the world, there is no consensus among surgeons for selection of reconstruction procedure out of Billroth I, Billroth II and Roux-en-Y gastrojejunostomy. The ideal procedure has to minimize postoperative morbidities and increase patients’ quality of life. While Billroth II is known to be easier and faster [15], bile reflux puts Billroth II under question when surgeons need to choose the surgical method. Roux-en-Y as an alternative for Billroth II has been associated with drawbacks such as longer duration and higher blood loss [4] which makes surgeons hesitate in selection of this method in a cluster of patients. Billroth II in combination with Braun anastomosis is suggested as an alternative for Roux-en-Y GJ which has been evaluated in our study and proved to be faster with less blood loss and similar outcomes and complications.

Our findings regarding the differences in surgery duration and intraoperative blood loss have been supported by previous reports in the literature. Cui et al. [4] have revealed surgery durations of 157.3 and 134.6 min in RY and B2B groups, respectively which show significant difference statistically but that study has not reported any difference in blood volume loss. Choi et al. [14] and Lirong et al. [16] have also reported similar findings. Tran et al. [17] have also reported that RY surgery has been slightly longer than B-II but blood loss has been lower in RY procedure. Jiang et al. [18] also reported no difference in terms of intraoperative blood loss between two procedures.

Early complications were the same between two groups. Re-admission and re-operation within 30 days after surgery were also similar. 30-day mortality was equal in both groups (∼2.3%). Wang et al. [19] have also reported that no significant difference in postoperative complications has been found between two groups. Jiang et al. [18] and Tran et al. [17] also supported these findings which were consistent with our study.

Theoretically, late and long-lasting complications after surgery can lead to malnutrition, longer hospital stays, re-admission, increased disease costs and declined quality of life. Additionally, BII-Braun can reduce specific complications such as loop syndrome or roux stasis syndrome and divert a remarkable volume of bile from remnant stomach away which can decrease development of complications. But practically, the reported findings are conflicting. No significant difference in late complications, reflux symptoms shown by GerdQ score, 90-day re-admission and 90-day mortality was noted. In endoscopic evaluation, all findings were the same except for food residue which was significantly more common in RY group and bile in stomach which was more common in B2B group. Consistent with our findings, Cui et al. [4] has reported that bile reflux was significantly more common in B2B group compared to RY group (28% vs 17.2%). Choi et al. [14] also reported higher levels of bile reflux and gastritis in B-II group. Several other reports have revealed similar results in terms of bile gastritis and reflux. In contrary to majority of studies, food residue in our study was more common in RY group. Cui et al. [4] reported no difference between two groups in terms of food residue and reflux esophagitis. Park et al. [20] also showed no difference between groups regarding food residue. In line with our study, Parthasarathy et al. [21] reported no significant difference for histopathologic findings between B2B and RY groups. Tran et al. [17] also reported similar 90-day mortality and the need for adjuvant therapy between groups which were consistent with our findings. Choi et al. [14] also reported one case of stricture in each group which was exactly the same as our study. Lirong et al. [16] indicated results in contrast to our findings. They reported that RY group significantly reduced gastritis, esophagitis, dumping symptoms and reflux symptoms (GerdQ score) which all were identical between two groups in our study.

Our study has several limitations which should be revised in future studies. The first limitation was relatively short period of follow-up. Longer follow-up periods can reveal enduring complications of the surgical reconstruction and ensure more appropriate selection of surgery technique in terms of consequent complications. The second limitation was low number of patients undergoing endoscopy. Routine endoscopy in future studies can help obtain more comprehensive results. The third limitation was that majority of patients had undergone surgery because of cancer and the impact of other indications on complications is not extensively studied. Larger sample sizes can help overcome this limitation in future studies.

## 5 Conclusion

Our findings showed that Billroth-II in combination to Braun anastomosis and Roux-en-Y gastrojejunostomy were comparable in terms of complications and mortality rates. Surgery duration and blood loss must be taken into account in critical cases. Food residue and bile in stomach are different between two procedures. Choice of surgery plan must be made with consideration of these factors.

## Provenance and peer review

Not commissioned, externally peer-reviewed.

## Ethical approval

The ethics committee of AJA University of Medical Sciences approved the study with the number IR.AJAUMS.REC.1398.092. The study was conducted in accordance with the Declaration of Helsinki and its later revisions.

## Please state any sources of funding for your research

No.

## Author contribution

**AS:** conception and design of the study, drafting the article, and approval of the final version of manuscript; **MV:** conception and design of the study, drafting the article, and approval of the final version of manuscript; **FK:** acquisition of data, critical revise, and approval of the final version of manuscript; **MFK:** acquisition of data, critical revise, and approval of the final version of manuscript**; MAP:** drafting the article, analysis and interpretation of data, and approval of the final version of manuscript**; FF:** conception and design of the study, drafting the article, analysis and interpretation of data, and approval of the final version of manuscript.

## Please state any conflicts of interest

No.

## Registration of research studies

1. Name of the registry: Iranian Registry of Clinical Trials
2. Unique Identifying number or registration ID: IRCT20130706013875N3
3. Hyperlink to your specific registration (must be publicly accessible and will be checked): https://www.irct.ir/trial/43309

## Guarantor

Farhad Fathi MD.

## Consent

“Written informed consent was obtained from the patient for publication of this study. A copy of the written consent is available for review by the Editor-in-Chief of this journal on request”.

## Annals of medicine and surgery

The following information is required for submission. Please note that failure to respond to these questions/statements will mean your submission will be returned. If you have nothing to declare in any of these categories then this should be stated.
